# Achaete-scute complex-like 2 regulated inflammatory mechanism through Toll-like receptor 4 activating in stomach adenocarcinoma

**DOI:** 10.1186/s12957-022-02722-y

**Published:** 2022-08-25

**Authors:** Enqi Zheng, Zhun Cai, Wangyong Li, Chuandou Ni, Qian Fang

**Affiliations:** Department of General Surgery, The First People’s Hospital of Wenling, No.333 south Chuan-an road, Chengxi street, Wenling, 317500 Zhejinag Province People’s Republic of China

**Keywords:** ASCL2, Stomach carcinoma, TLR4, Inflammation

## Abstract

**Background:**

To investigate the role of achaete-scute complex-like 2 (ASCL2) in stomach adenocarcinoma (STAD), we analyze whether ASCL2 suppression could retard cancer development and further observe the relevance between ASCL2 and inflammation via Toll-like receptor 4 (TLR4) activation in STAD, both in vitro and in vivo.

**Methods:**

Proliferation, development, inflammation, and apoptosis in STAD are observed using sh-ASCL2 lentivirus via TLR4 activation in vitro and in vivo. The relationship between ASCL2 and inflammation is analyzed. Western blotting of ASCL2 with the target protein of immune-associated cells is performed. The prognosis of STAD and associated ASCL2 mutation are analyzed.

**Results:**

The ASCL2 level in STAD tumor tissues is increased, compared to normal tissues, and brings a worse prognosis. The ASCL2 shows a negative correlation with inflammation, and TLR4 reveals a positive correlation with gastric cancer. ASCL2 expression is high in MGC803 cells. Sh-ASCL2 could reduce STAD development by decreasing proliferation, tumor volume, and biomarker levels and increasing apoptosis in vitro and in vivo. The inflammatory role of ASCL2 is regulated through TLR4 activation. ASCL2 levels may be related to CNTNAP3, CLIP1, C9orf84, ARIH2, and IL1R2 mutations; positively correlated with M2 macrophage and T follicular helper cell levels; negatively correlated with neutrophil, dendritic cell, monocyte, CD8 T cell, and M1 macrophage levels; and involved in STAD prognosis.

**Conclusions:**

The ASCL2 may adjust inflammation in STAD through TLR4 activation and may be associated with related immune cells. ASCL2 is possibly an upstream target factor of the TLR4 signaling pathway.

## Background

Gastric cancer is one of the most common upper gastrointestinal cancers, worldwide, and it remains a major cause of malignant disease-associated morbidity and mortality [1]. Chronic gastritis is due to an infection of *Helicobacter pylori* (*H. pylori*), and it has been reported that inflammatory response-related genes are linked to the etiology of gastric cancer [2].

The pathogenesis of gastric cancer is associated with many factors including genetic changes and the immune environment. Further progress of gastric mucosa variation possibly ascribes evasive mechanisms induced by virulence factors such as bacteria [3]. Epithelial cells, neutrophils, macrophages, and other immune cells can infiltrate the gastric mucosa and release a series of anti-microbial compounds, such as interleukin (ILs) [4]. Activation of Toll-like receptor 4 (TLR4) and nuclear factor kappa B (NF-κB) pathways leads to inflammatory functionality and facilitates the release of pro-inflammatory chemokines [5].

Tumor cells exploit immune checkpoint pathways to reduce immune response and then lead to escape immune surveillance. Consequently, the discovery of therapeutic antibodies targeting immune checkpoints has been a considerable breakthrough, particularly for cancer treatment [6]. Preclinical studies have shown that antitumor immunity could improve immunotherapies for malignant tumors [7]. Hence, searching for significant inflammation markers of the cell of origin and its growth regulation will be crucial to the treatment and prevention of gastric cancer.

Achaete-scute complex-like 2 (ASCL2) is a member of the basic helix-loop-helix family of transcription factors, binds to the E-box (5′-CANNTG-3′), and stimulates the transcription [8]. Recent studies have reported that ASCL2 may contribute to the pathogenesis of autoimmunity and immune resistance in Sjögren syndrome, colorectal cancer, and lung adenocarcinoma [9]; however, the mechanism underlying the role of the ASCL2 in inflammation in stomach carcinoma has not been explained.

Not only can the function of TLR4 be explained as a pivotal regulator in innate and acquired immunity but can also be regarded as linking to the occurrence and development of multifarious cancers [10]. Nevertheless, in stomach carcinoma, the potential role of ASCL2 in inflammation via TLR4 activation or its pathways has not been completely clarified.

The study aims to investigate the potential role and function of ASCL2 in STAD, then apply ASCL2 knockdown lentivirus (sh-ASCL2) to enhance apoptosis, inflammatory progress, and tumor development in vivo and in vitro. Also, TLR4 agonists were used to induce the TLR4 expression and reverse the progress of inflammation by regulating ASCL2. Our findings indicate that ASCL2 could potentially regulate inflammation in STAD via TLR4 activation, which may offer a novel strategy for STAD treatment through the inflammation-associated mechanism.

## Methods

### Human stomach carcinoma specimens

We select 22 patients, who have been pathologically diagnosed with stomach carcinoma by the gastrointestinal surgery and pathology department of our hospital from May 2019 to May 2021, and obtain adjacent paracarcinoma tissues as controls. All patients provide informed consent, and the experimental design is approved by the Ethics Committee of The First People’s Hospital of Wenling.

### Bioinformatics analysis

ASCL2 expressed in different cancers assayed by the TIMER2.0 website (http://timer.cistrome.org/) and the Gene Expression Profiling Interactive Analysis (GEPIA) website (https://gepia.cancer-pku.cn/) were assayed. The survival assay was developed using the Kaplan–Meier plotter website (http://kmplot.com/analysis/index.php?p=service&cancer=gastric). Mutations in STAD were developed using the cBioPortal website (http://www.cbioportal.org). The data from 478 sample datasets, named as stomach adenocarcinoma (TCGA, Firehose Legacy), are collected.

### Cell lines and antibodies

The cell lines used are as follows: GES-1 (CL-0563, Procell, Wuhan, China), AGS (CL-0022, Procell, Wuhan, China), SGC-7901 (CL-0206, Procell, Wuhan, China), MGC803 (ML-CS-0276, ATCC, VA, USA), MKN-45 (CL-0292, Procell, Wuhan, China), and SNU-1 (CL-0474, Procell, Wuhan, China).

The following antibodies are used: anti-ASCL2 (20, R&D Systems, AF6539, Minneapolis, USA); anti-ki67 (ab15580, Abcam, Cambridge, USA); anti-MPO (ab6208670, Abcam, Cambridge, USA); anti-CD1c (sc-390980, Santa Cruz Biotechnology, Heidelberg, Germany); anti-CD206 (ab64693, Abcam, Cambridge, USA); anti-CD163 (ab182422, Abcam, Cambridge, USA); anti-CD8a (sc-1177, Santa Cruz Biotechnology, Heidelberg, Germany); anti-CD109 (sc-271085, Santa Cruz Biotechnology, Heidelberg, Germany); anti-iNOS (ab178945, Abcam, Cambridge, USA); anti-CEA (ab207718, Abcam, Cambridge, USA); anti-CA199 (ab3982, Abcam, Cambridge, USA); anti-c-Met (ab51067, Abcam, Cambridge, USA); anti-TLR4 (ab13556, Abcam, Cambridge, USA); anti-NF-κB (BM3940, Boster, Wuhan, China); anti-IL-1β (ab2105, Abcam, Cambridge, USA); anti-IL-18 (ab207323, Abcam, Cambridge, USA); and anti-β-actin (M01263-2, Boster, Wuhan, China). The following secondary antibodies conjugated with horseradish peroxidase are used: anti-rabbit IgG (H + L) (AS014, ABclonal, Wuhan, China) and anti-mouse IgG (H + L) (AS003, ABclonal, Wuhan, China). Sparstolonin B (HY-116213, MedchemExpress, Dallas, USA) is also used.

### Cell culture

GES-1, a human normal gastric mucosa cell line, and AGS, SGC-7901, MGC803, MKN-45, and SNU-1, stomach carcinoma cell lines, are used. GES-1 cells are maintained in RPMI-1640 (Thermo Fisher Scientific, Inc., MA, USA) with 10% fetal bovine serum at 37 °C in a humidified chamber supplemented with 5% CO_2_. Cells are maintained and cultured, as described in reference [11].

### ASCL2 knockdown lentivirus administration

The ASCL2 knockdown lentivirus (sh-ASCL2) and control lentivirus are designed and chemically synthesized (GenePharma Corporation, Shanghai, China) and stored at – 80 °C. The administration of rectal carcinoma cells by lentivirus is according to well-established guidelines [12]. The sequences of the ASCL2 lentivirus are shown in Table 1.

**Table 1:** The name and sequence of ASCL2 lentivirus

Name	Sequence
sh-ASCL2-301	5′-CGTCCCTGTCGGCTGCGCTG-3′
sh-ASCL2-611	5′-GGGCTGAGGCCGCAGGCCGT-3′
sh-ASCL2-782	5′-CTGAGTCCTGCGGAGCGCGA-3′
Negative control	5′-CCACCCCCCGCACCGCCGAC-3′
control-shASCL2	5′-CCTGGGGAGTTGCCTGGCGG-3′

Then, four groups (*n* = 6 in each group) are randomly divided: Con: untreated MGC803 cells or mice; sh-ASCL2: MGC803 cells or mice treated with sh-ASCL2; control-shASCL2: MGC803 cells or mice treated with the ASCL2 control lentivirus; and Spa. + sh-ASCL2: MGC803 cells or mice treated with sh-ASCL2 and managed with sh-ASCL2 as well as sparstolonin B (10 μg in vitro or 10 mg/kg in vivo).

### Propidium iodide (PI) staining [13]

Cells are subjected to a PI-Hoechst assay (40755ES64, Qcbio Science&Technologies Co., Ltd., Shanghai, China) and treated with different concentrations of Rh1 for 24 h. After the treatment, the cells are washed with PBS before being stained with PI (10 μM) at 37 °C for 10 min. The cells are then washed with PBS, stained with Hoechst (5 μg/ml) for 10 min, and fixed in 4% formaldehyde for 10 min.

### Animals

Healthy male Balb/c mice (2 days or 4 weeks old) are housed under specific pathogen-free (SPF) conditions with food and reverse osmosis purified water (supplied by Liaoning Changsheng Biotechnology Co., Ltd., Shenyang, Liaoning, China). Mice are cared for in strict accordance with the Guide for the Care and Use of Laboratory Animals (NIH Publication No. 85–23, revised 1996), and the experimental design is approved by the Ethics Committee of Liaoning Changsheng Biotechnology Co., Ltd.

### Stomach carcinoma mouse model

Male Balb/c mice (4 weeks old) are subcutaneously injected with MGC803 cells, as described previously [12, 13]. When the mice exhibit rapid weight loss (> 20%) or metastatic burden, they are sacrificed.

### Immunohistochemical (IHC) staining

Four-micrometer sections are used after xylene deparaffinization, ethanol dehydration, and antigen retrieval. Endogenous peroxidase activity is quenched by incubation in hydrogen peroxide solution (Peroxidazed 1, PX 968, Biocare Medical, Pacheco, CA, USA) for 5 min. Sections from each sample are incubated overnight with the primary antibodies against the ASCL2 and ki-67. Streptavidin–biotin-peroxidase staining kit (Goldenbridge Biotechnology Co., China) is measured to signal.

### Western blotting

Proteins are homogenized in RIPA lysis buffer (Beyotime, Shanghai, China), to which two kinds of protease inhibitor cocktails (MCE, HY-K0022) have also been added. Total protein extracted using RIPA reagent from each sample is subjected to 12% SDS–polyacrylamide gel electrophoresis and transferred to nitrocellulose membranes. The proteins are separated on polyacrylamide gels and finally detected with ChemiDoc™ Touch Imaging System (Bio-Rad, CA, USA).

### TUNEL staining [14]

Tissue fractions are maintained onto the chamber sections and examined with TUNEL staining utilizing In Situ Cell Death Detection Kits in accordance with the supplier’s specification. Images are captured using an Olympus BX41 fluorescence microscope. Image quantification is analyzed using a TUNEL assay (ab66108, Abcam, Cambridge, UK), as described previously.

### MTT

MTT assays are performed to assess cell proliferation as described [15]. The cells are seeded in 96-well plates at a density of 1000 cells per well with 5 replicate wells at 48 h post-siRNA transfection. Cell proliferation is measured using an MTT assay at 0, 24, 48, 72, 96, 120, and 144 h after seeding. Next, 150 μl dimethylsulfoxide is added per well to dissolve the purple formazan. The absorbance is measured at a wavelength of 490 nm. To avoid the affection of cell proliferation on cell motility, cells for transwell are cultured in a low-serum concentration (0.2%).

### Statistical analysis

The mean ± standard deviation values are calculated and statistical analysis is performed using one-way or two-way ANOVA, where *P*-values < 0.05 are considered statistically significant. Holm-Sidak test is used to analyze multiple comparisons.

## Results

### Bioinformatics analysis of the ASCL2 in STAD using TCGA, TIMER, and Kaplan–Meier plotter system

**Fig. 1:**
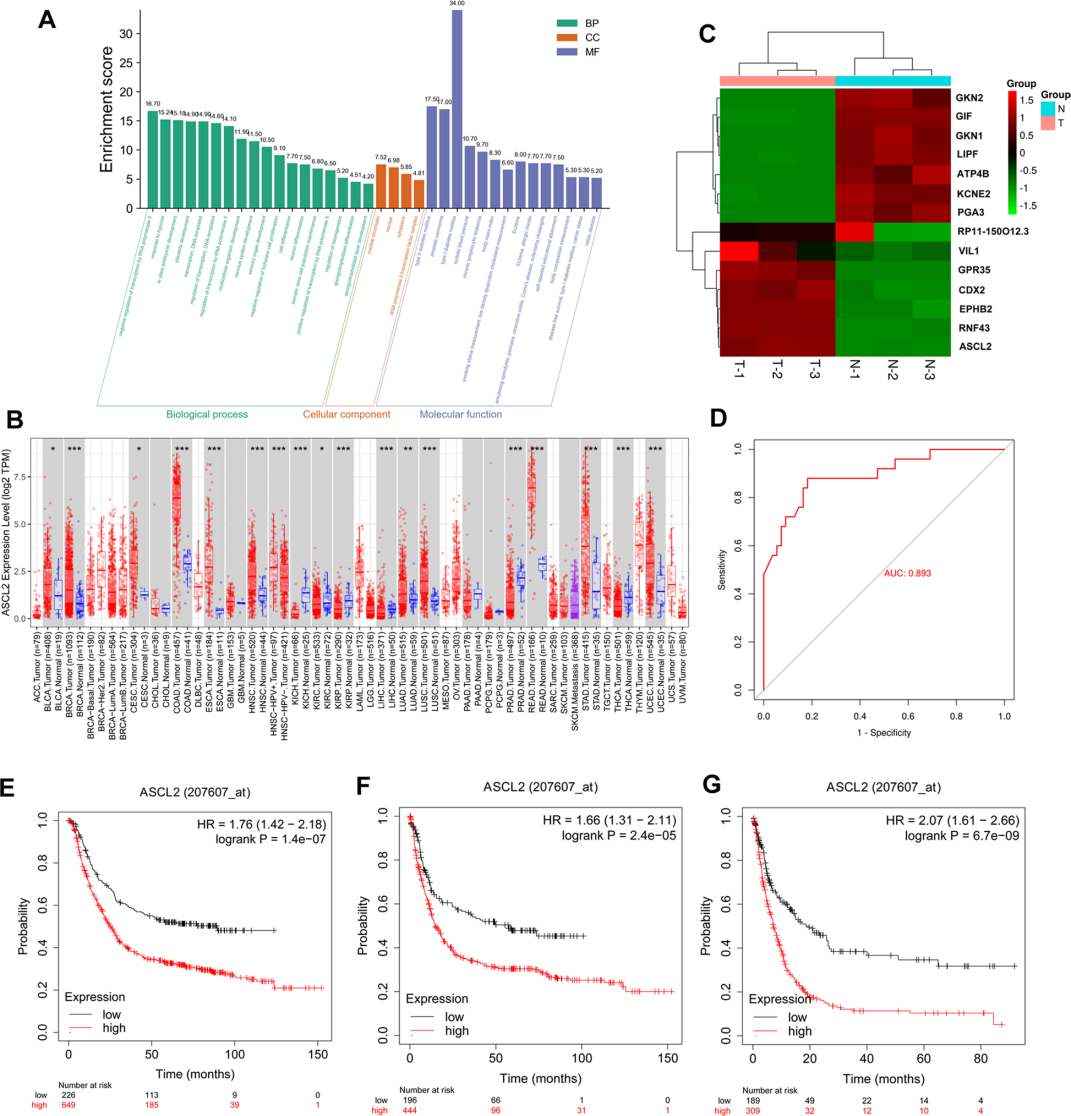
**A** GO enrichment assay contains BP, CC, and MF of ASCL2 in STAD. **B** Level of ASCL2 in different cancers. **C** Heatmap of similar genes with ASCL2 in STAD. **D** ROC curve of ASCL2 in STAD. **E** OS, **F** FPS, and **G** PPS analyses in STAD patients

The results regarding the role of the ASCL2 in STAD, predicted using TCGA, TIMER, and Kaplan–Meier plotter system, are shown in Fig. 1. Figure 1A includes the Gene Ontology (GO) of the ASCL2 regarding the biological processes (BP), cellular components (CC), and molecular functions (MF), and the expression of the ASCL2 was quantified in different kinds of cancers (Fig. 1B). The level of ASCL2 in STAD tumor tissues is increased (*P* < 0.05). The heatmap of genes similar to the ASCL2 in STAD is shown in Fig. 1C. The receiver operating characteristic (ROC) curve of the ASCL2 in STAD is shown in Fig. 1D. Based on the analyses of the overall survival (OS), first progressive survival (FPS), and post-progressive survival (PPS), we can easily infer that when the expression of the ASCL2 isoform is high (Fig. 1E–G), high levels of the ASCL2 STAD patients have a worse prognostic outcome (*P* < 0.05).

### Different levels of the ASCL2 expression in different STAD tissues or cell lines

**Fig. 2:**
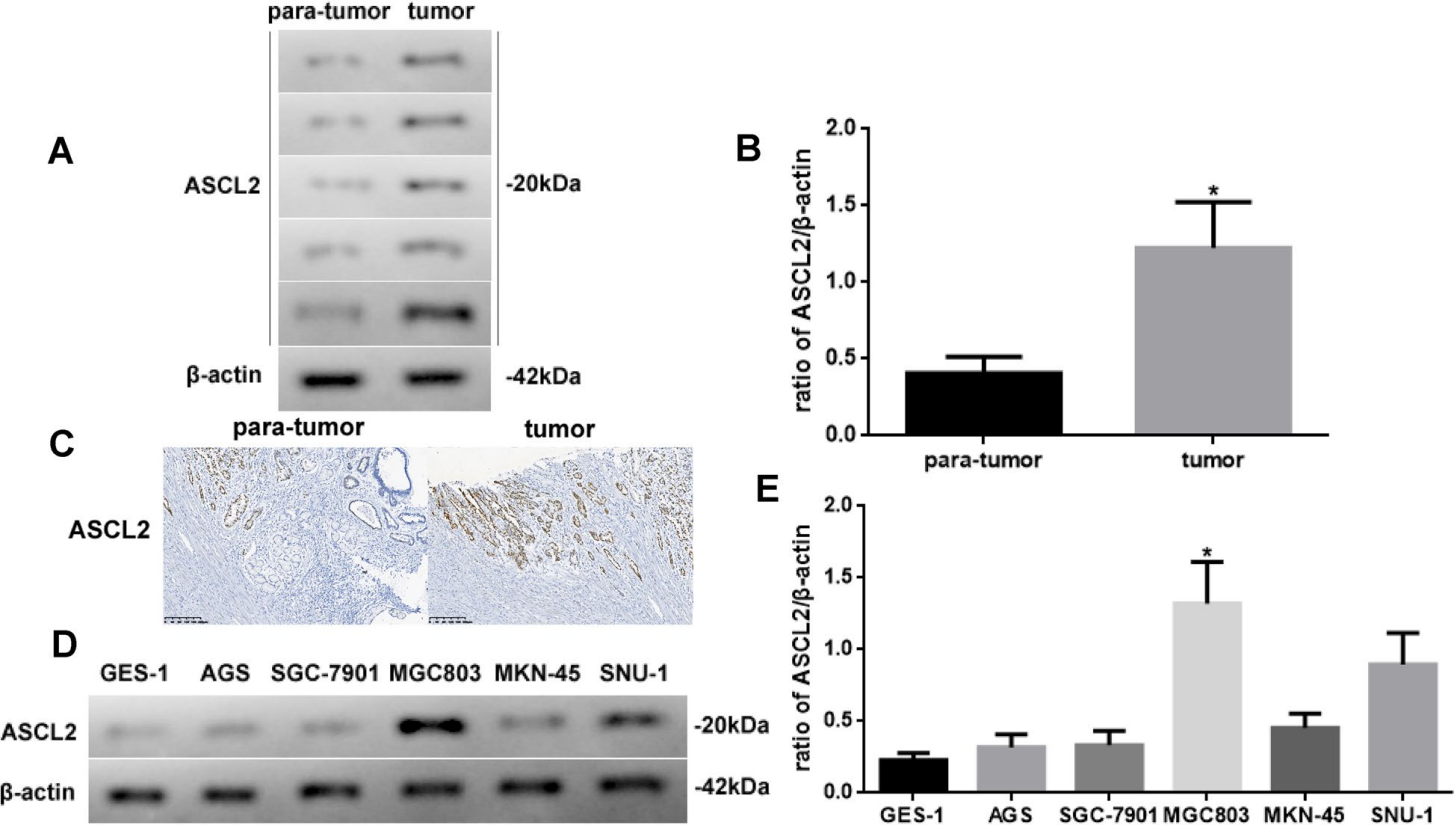
**A** ASCL2 expressed and **B** quantificated in different tissues. **C** IHC for ASCL2 (× 100). **D** ASCL2 expressed and **E** quantificated in different cell lines. Protein levels were normalized to β-actin (tumor vs. para-tumor, and MGC803 vs. other cells, *n* = 6, *P < 0.05)

As the *ASCL2* was predicted to be a STAD-related gene using bioinformatics analysis, we observe the level of the ASCL2 in different tissues or cell lines using western blotting and IHC staining (Fig. 2). The expression of the ASCL2 in tumor tissues is induced (Fig. 2A, B, *P* < 0.05). Besides, the ASCL2 expression occurs in the nuclei, as observed in the IHC-stained sections in Fig. 2C. In addition, the ASCL2 expression in MGC803 cells is mostly significantly induced (Fig. 2D, E, *P* < 0.05). Moreover, we use MGC803 cells for the follow-up experiments.

### The ASCL2 knockdown weakens the development of STAD in vitro

**Fig. 3:**
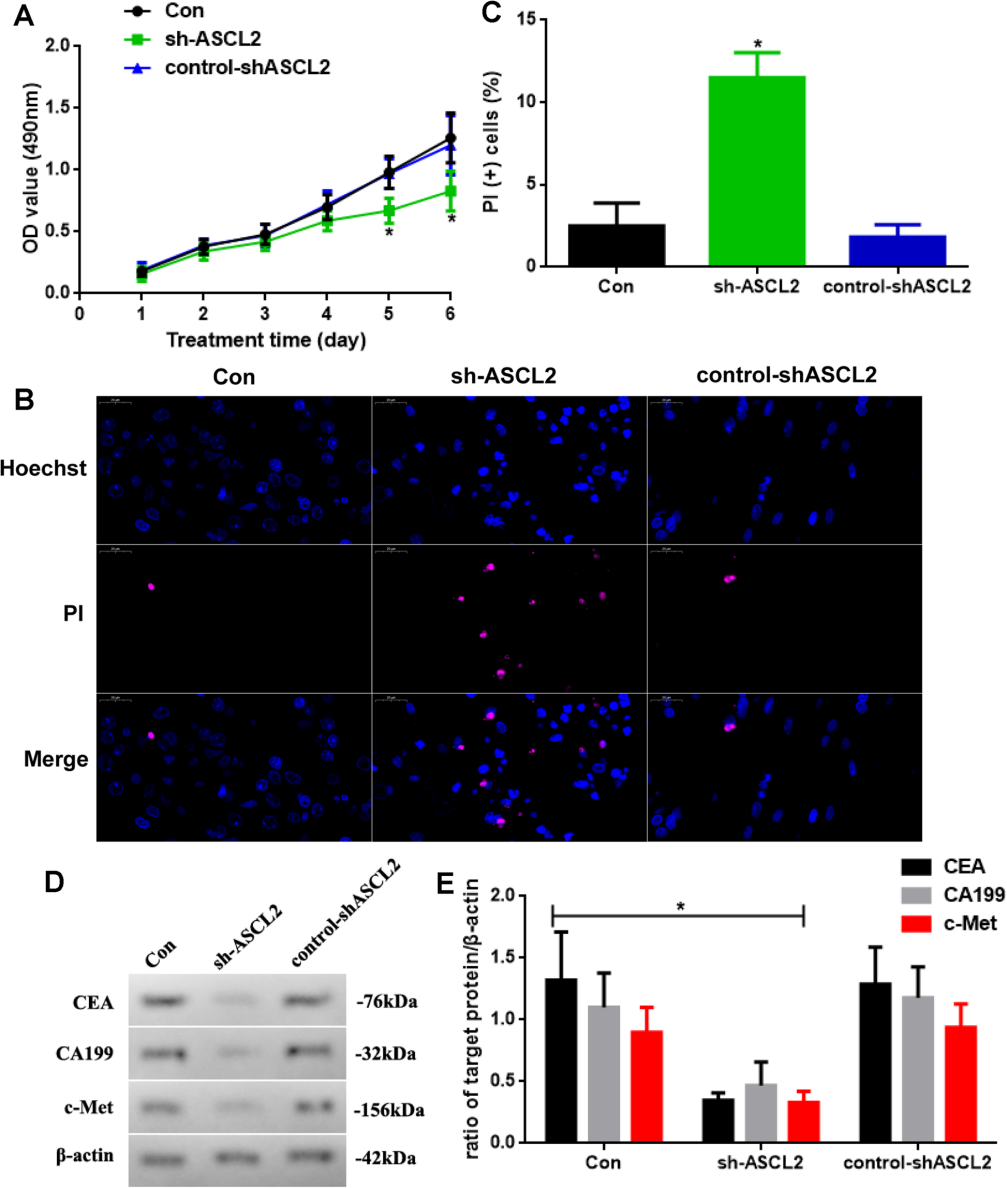
**A** Proliferation of cells in different groups. **B** PI-Hoechst staining (× 400) and **C** PI ( +) cell assay. **D** CEA, CA199, and c-Met expressed and **E** quantificated (sh-ASCL2 vs. other groups, *n* = 6, *P < 0.05)

As the expression of the ASCL2 in the tumor tissues is significantly increased, we hypothesized that the ASCL2 is probably related to the stomach carcinoma progression. Thus, we block the ASCL2 expressed by using sh-ASCL2 lentivirus in Fig. 3. We test the proliferation in different cells. The OD values in the sh-ASCL2 group at days 5 and 6, which are detected using MTT, are significantly decreased (Fig. 3A). To further investigate the function of the ASCL2 in apoptosis, PI-Hoechst staining is observed (Fig. 3B), and apoptosis level in the sh-ASCL2 group is enhanced (Fig. 3C, *P* < 0.05). In addition to this, we also detect the levels of tumor biomarkers (Fig. 3D). CEA, CA199, and c-Met in the sh-ASCL2 group are reductive (Fig. 3E, *P* < 0.05).

### The ASCL2 knockdown weakens the development of STAD in vivo

**Fig. 4:**
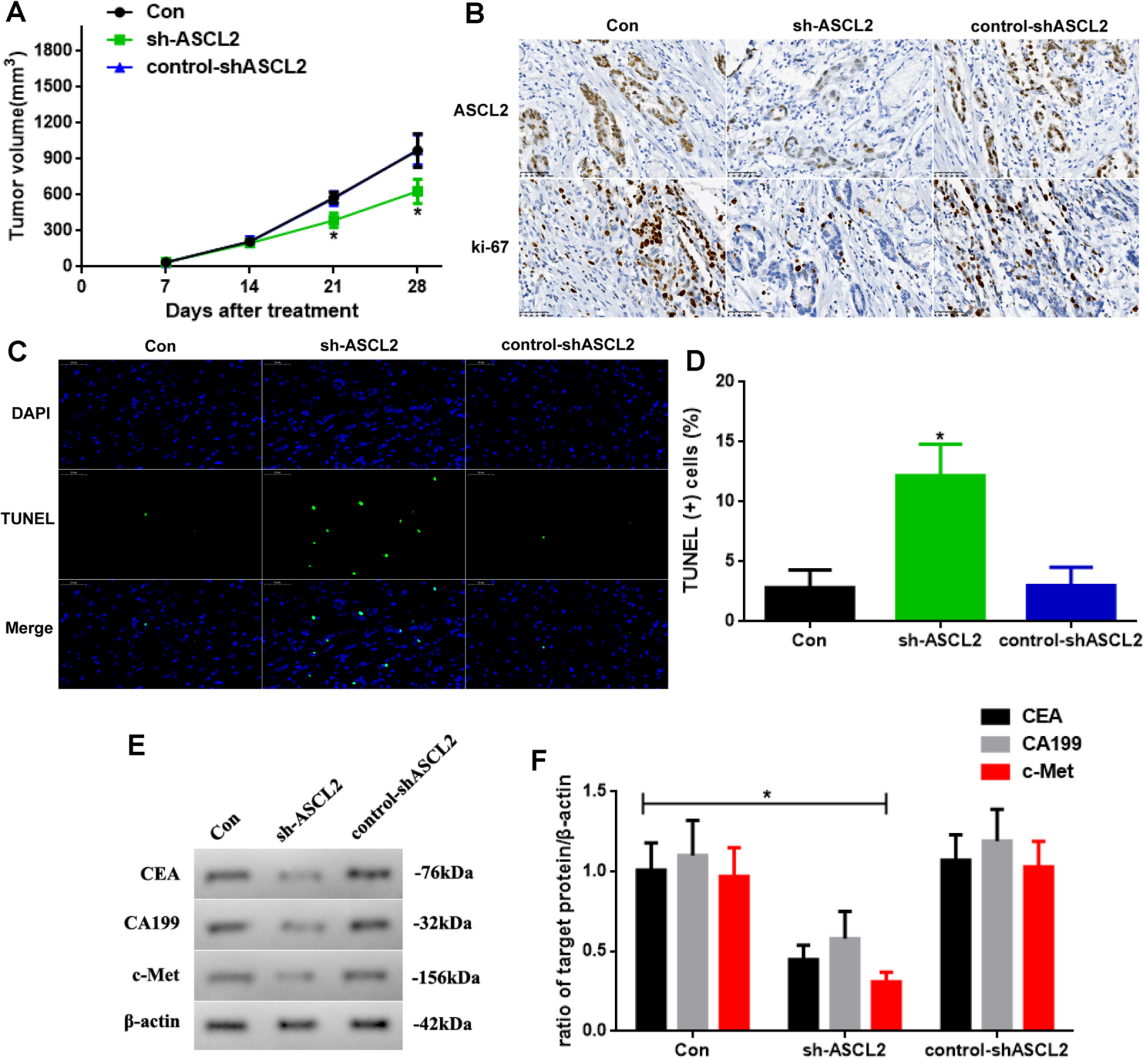
**A** Tumor volume of different mice. **B** IHC for ASCL2 and ki-67 index (× 400). **C** TUNEL-DAPI staining (× 400) and **D** TUNEL ( +) cell assay. **E** CEA, CA199, and c-Met expressed and **F** quantificated (sh-ASCL2 vs. other groups, *n* = 6, *P < 0.05)

As the ASCL2 knockdown weakens the development of STAD in the previous outcomes, we also test it in vivo (Fig. 4). First, we observe the tumor volume in different mice. The volumes at days 21 and 28 in sh-ASCL2 mice are significantly reduced (Fig. 4A, P < 0.05). Second, the expression of the ASCL2 and ki-67 are detected using IHC staining, as shown in Fig. 4B. The ASCL2 expression and the ki-67 index are reduced by the ASCL2 knockdown. Third, TUNEL assay is detected (Fig. 4C). The apoptosis levels in sh-ASCL2 mice are enhanced (Fig. 4D, *P* < 0.05). At last, tumor biomarkers of STAD are also tested (Fig. 4E). CEA, CA199, and c-Met in the sh-ASCL2 group are reductive (Fig. 4F, *P* < 0.05), which is consistent with the previous outcomes.

### Bioinformatics analysis of the ASCL2 with inflammation in STAD using TCGA

**Fig. 5:**
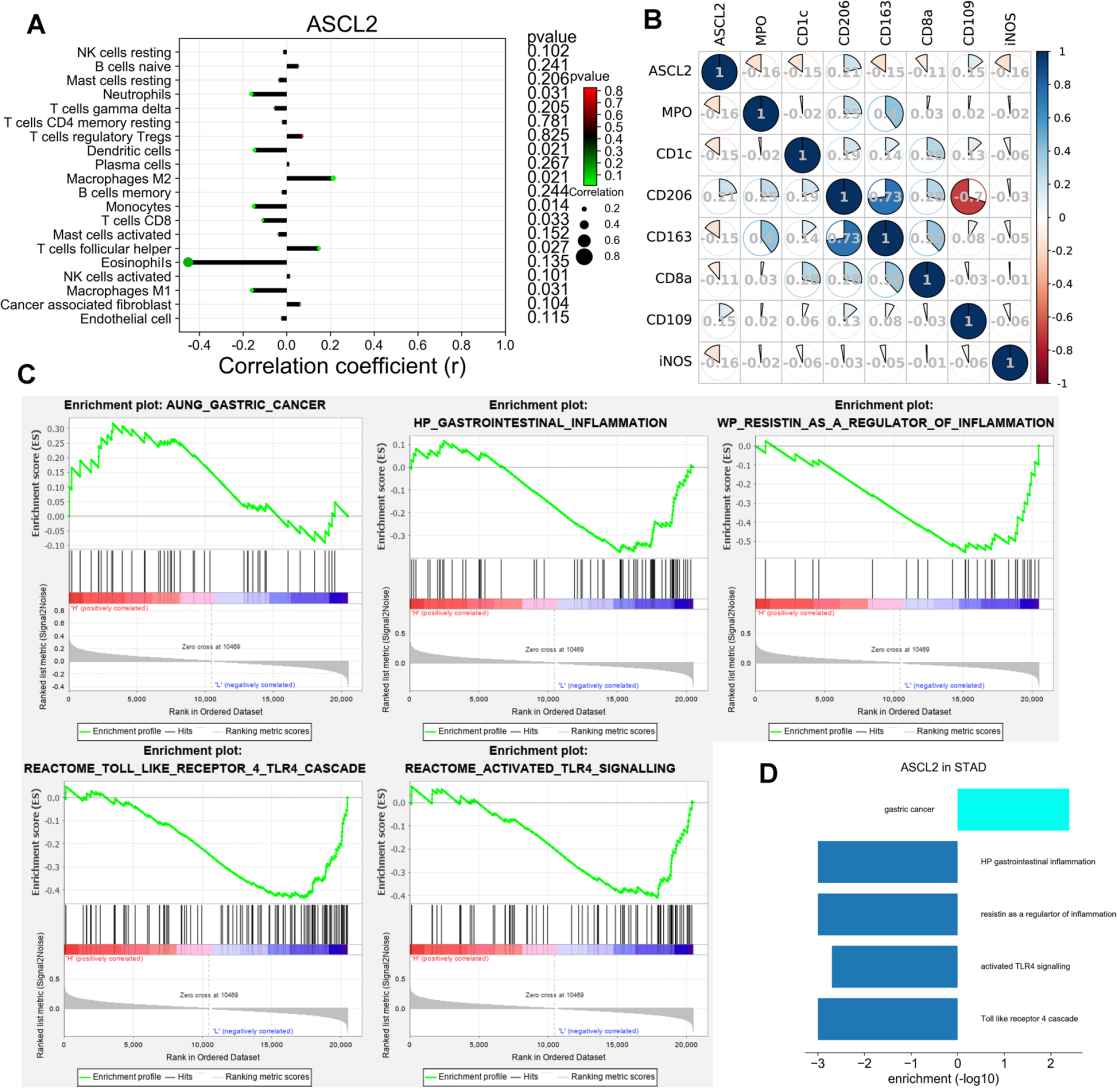
**A** Correlation of ASCL2 with immune-associated cells in STAD. **B** Correlation of ASCL2 with target protein of macrophage M2, T cell follicular helper, neutrophils, dendritic cells, monocytes, T cells CD8, and macrophage M1. **C**, **D** GSEA assay of ASCL2 with gastric cancer, inflammation, and TLR4 in STAD

To further investigate the function of the ASCL2 and inflammation in STAD, we use enrichment analysis. The correlation coefficient of the ASCL2 with immune-associated cells in STAD is shown in Fig. 5A. We can easily infer that the ASCL2 reveals a positive correlation with the levels of M2 macrophages and T follicular helper cells and shows a negative correlation with the levels of neutrophils, dendritic cells, monocytes, CD8 T cells, and M1 macrophages. The correlation coefficient between the ASCL2 level and the target protein levels of M2 macrophages, T follicular helper cells, neutrophils, dendritic cells, monocytes, CD8 T cells, and M1 macrophages is shown in Fig. 5B. The ASCL2 reveals a positive correlation with CD206 and CD109 levels and shows a negative correlation with MPO, CD1c, CD163, CD8a, and iNOS levels. The ASCL2 shows a negative correlation with *H. pylori*-induced gastrointestinal inflammation, uses resistin as a regulator of inflammation, activates TLR signaling and TLR4 cascade, and positively correlates with gastric cancer by GSEA (Fig. 5C, D).

### The ASCL2 knockdown can regulate the target protein of immune-associated cells of stomach carcinoma in vivo

**Fig. 6:**
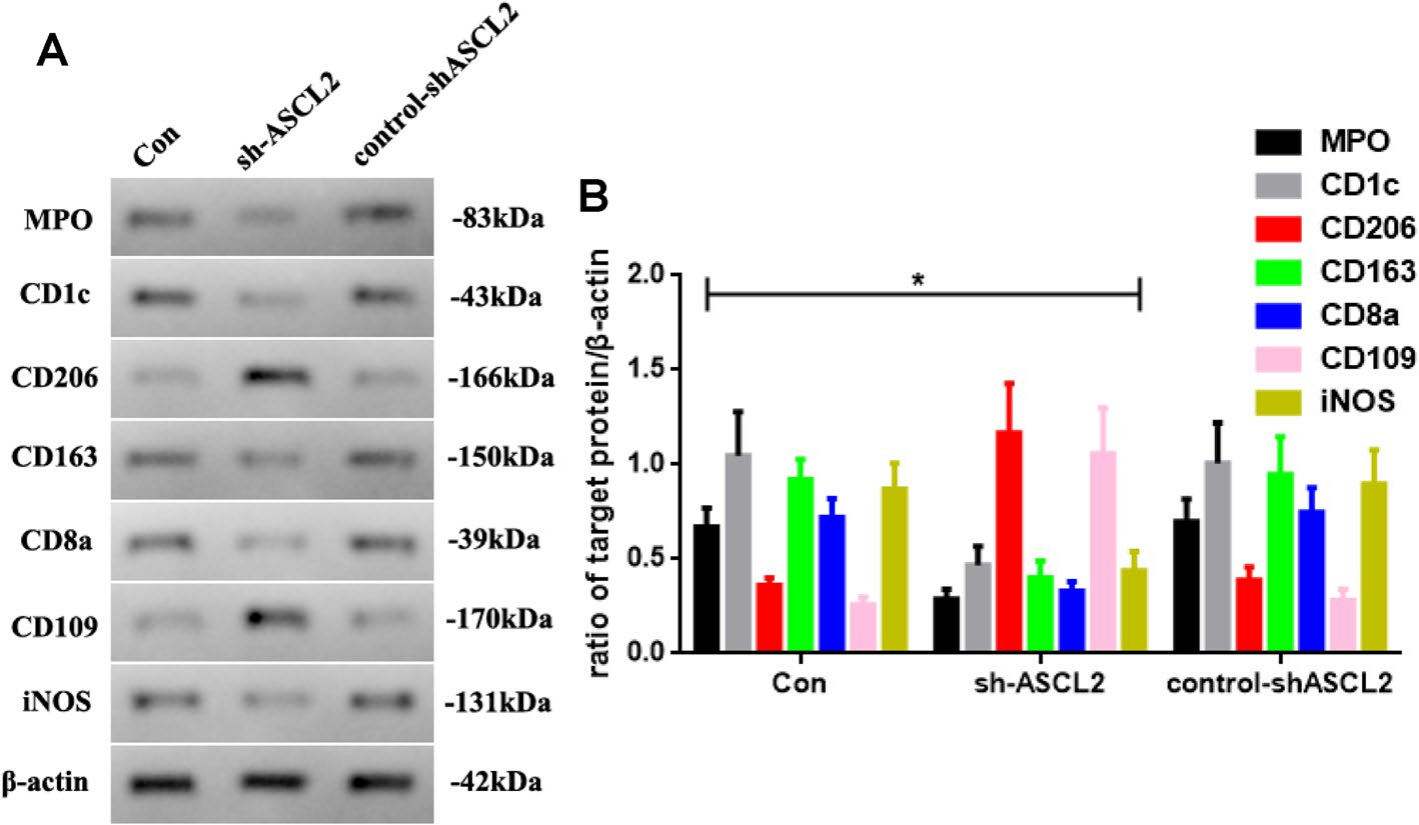
**A** MPO, CD1c, CD206, CD163, CD8a, CD109, and iNOS expressed in different groups, and **B** quantificated (sh-ASCL2 vs. other groups, *n* = 6, *P < 0.05)

As the ASCL2 can possibly be an immunoregulatory gene in STAD in the previous outcomes, we detect the target protein of immune-associated cells using western blotting in vivo. In Fig. 6A, MPO, CD1c, CD163, CD8a, and iNOS in the sh-ASCL2 group is reductive (*P* < 0.05). Moreover, the levels of CD206 and CD109 are increased compared with other groups (Fig. 6B).

### The ASCL2 knockdown increases the inflammatory level, which is regulated through TLR4 in vitro and in vivo

**Fig. 7:**
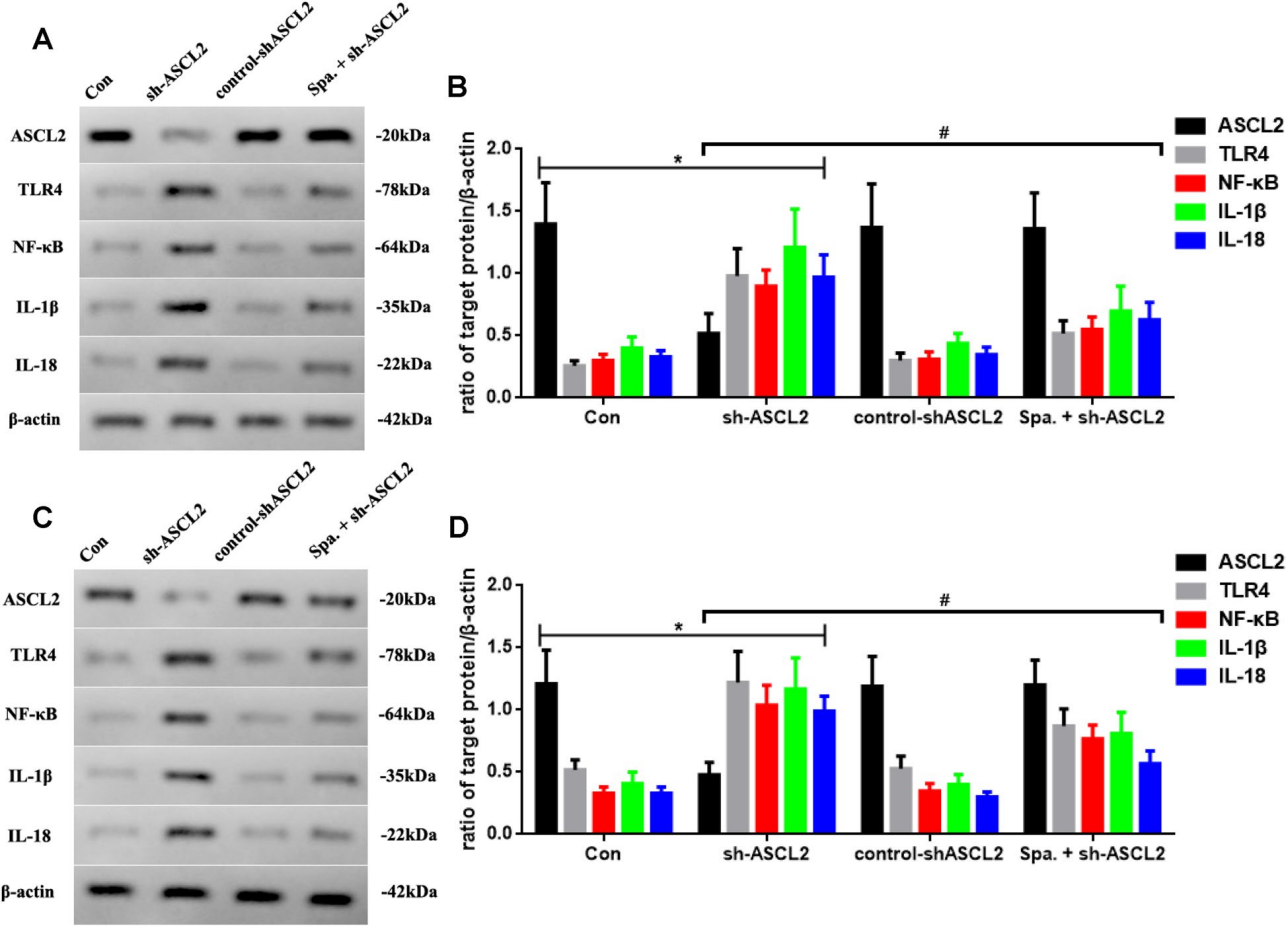
**A** ASCL2, TLR4, NF-κB, IL-1β, and IL-18 expressed in different groups, and **B** quantificated in vitro. **C** ASCL2, TLR4, NF-κB, IL-1β, and IL-18 expressed in different groups and **D** quantificated in vivo (sh-ASCL2 vs. Con, *n* = 6, *P < 0.05; Spa. + sh-ASCL2 vs. sh-ASCL2, *n* = 6,.^#^*P* < 0.05)

Next, we use sparstolonin B to activate the TLR4 signal for confirming the tumor-regulated effect of the ASCL2, following which we observe the changes (Fig. 7). Sparstolonin B increases the levels of TLR4, NF-κB, IL-1β, and IL-18, after the ASCL2 knocking down (Fig. 7A, B, *P* < 0.05). Besides, sparstolonin B cannot significantly change the level of the ASCL2. Similarly, the same in vivo outcomes are shown in Fig. 7C, D.

### Further bioinformatics analysis of the ASCL2 in STAD patients using cBioPortal and GEPIA system

**Fig. 8:**
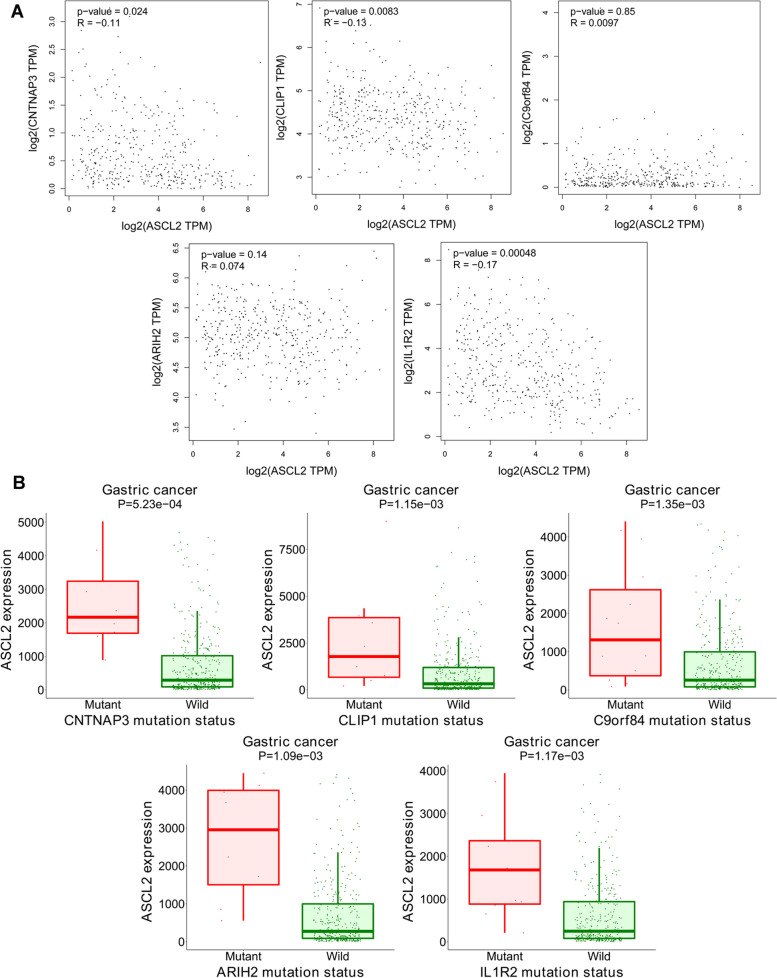
**A** Correlation analysis between ASCL2 with CNTNAP3, CLIP1, C9orf84, ARIH2, and IL1R2 in STAD. **B** Expression of ASCL2 in CNTNAP3, CLIP1, C9orf84, ARIH2, and IL1R2 mutant and wild-type STAD patients

In STAD, correlation analysis using the GEPIA system shows that the ASCL2 reveals a positive correlation with C9orf84 and ARIH2 levels and a negative correlation with the levels of CNTNAP3, CLIP1, and IL1R2 (Fig. 8A). In the ASCL2 high expression group, the number of *CNTNAP3*, *CLIP1*, *C9orf84*, *ARIH2*, and *IL1R2* mutations is significantly increased, conformably the same with the results in Fig. 8B. This may be related to *CNTNAP3*, *CLIP1*, *C9orf84*, *ARIH2*, and *IL1R2* mutations and involved in stomach carcinoma prognosis.

**Fig. 9:**
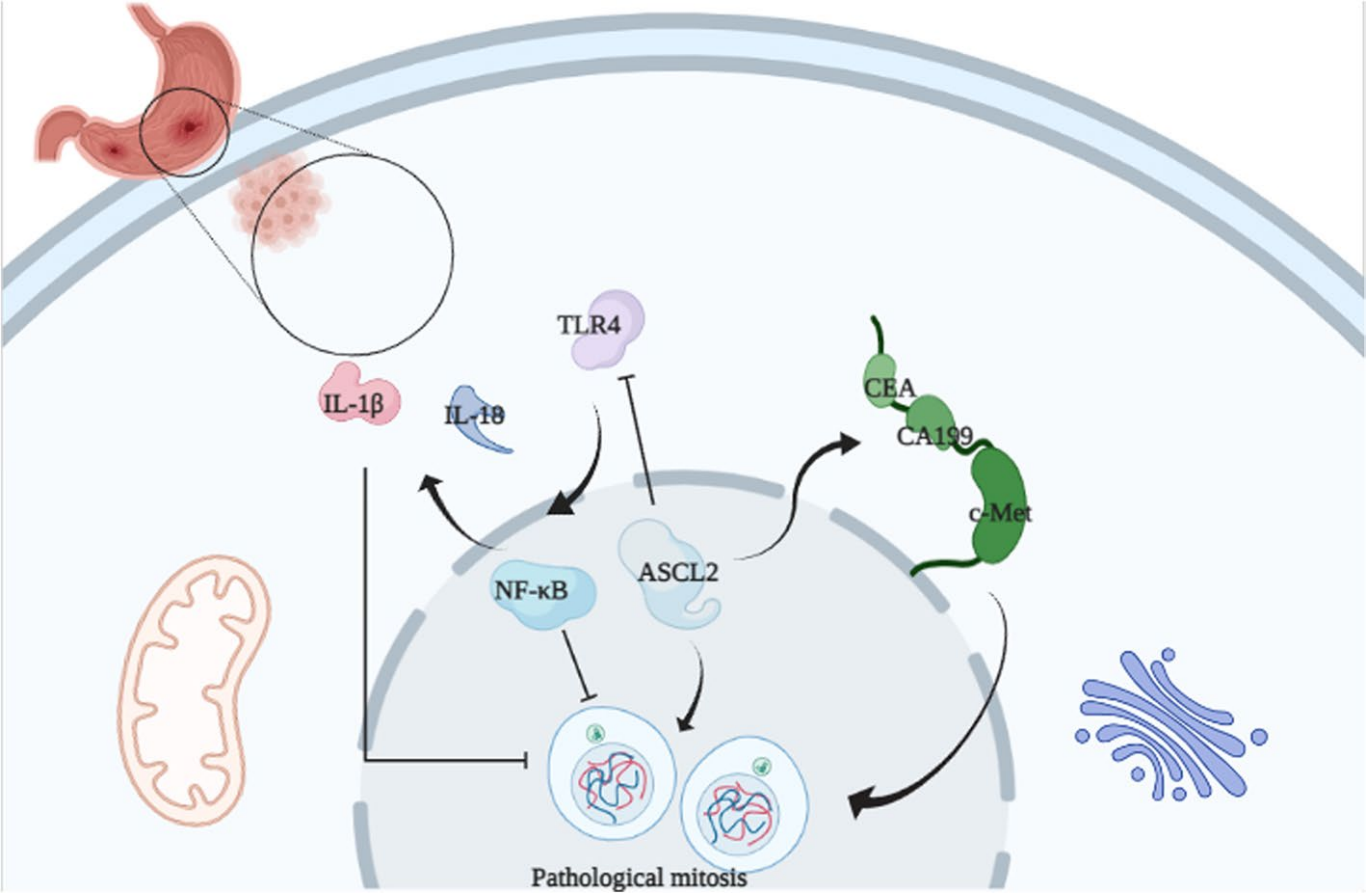
ASCL2 mediated inflammation in STAD. Activating TLR4 may recede the suppressive inflammatory factors expression, including NF-κB, IL-1β, and IL-18 by ASCL2 regulation, and induce the inflammatory injury to tumor cells

The ASCL2 mediated inflammation in STAD is shown in Fig. 9. Activating TLR4 may recede the suppressive inflammatory factors expression, including NF-κB, IL-1β, and IL-18 by the ASCL2 regulation, and induce the inflammatory injury to tumor cells, thereby being involved in the prognosis of this disease.

## Discussion

Gastric carcinoma is one of the most aggressive types of cancer, and it ranks fifth and third with incidence and cancer mortality, respectively [16]. As it exhibits a prolonged asymptomatic condition and a high recurrence rate, it is very challenging to treat. It has become widely accepted that inflammation is a driving force behind cancers; however, the existing immunotherapies show a limited utility in clinical practice [17]. Therefore, novel agents with different inflammatory mechanisms are needed.

The ASCL2 is primordially demonstrated to be involved in the determination of the neuronal precursors in the peripheral nervous system and central nervous system [8]; however, ASCL2 was recently verified to be related to inflammation in cancer, such as colorectal cancer, lung adenocarcinoma, and breast cancer [18]. In our study, we first use GO assay for ASCL2 to explore its related biological processes, cellular components, and molecular functions, as they have been rarely mentioned in oncology.

The expression of ASCL2 in STAD tumor tissues is increased, which indicates ASCL2 could possibly be an oncogene in STAD. Regarding the analyses of survival, high ASCL2 levels in STAD patients mean a worse prognostic outcome. All these results indirectly prompt that ASCL2 might be related to STAD progress.

Based on the previous results of ASCL2 in STAD, we observe the different levels of ASCL2 in different tissues or cell lines. The ASCL2 expression is induced in tumor tissues or cells, which is coincident with the previous analysis. Interestingly, the ASCL2 is expressed in the nuclei of cells in STAD.

For further research, we hypothesize that the ASCL2 is correlated with stomach carcinoma progression. Thus, we use sh-ASCL2 to block the ASCL2 expression. In our study, sh-ASCL2 can inhibit STAD development by increasing apoptosis and reducing proliferation, tumor volume, and biomarker levels. These results demonstrate that the ASCL2 is potentially able to adjust apoptosis and cancer progression in STAD.

Therefore, inflammation plays a dual function by reducing tumor progression rate [19] and deactivating cell-mediated immune-suppressive mechanisms in cancer, thus being involved in cancer immunotherapy [20]. To further explore the ASCL2 in cell-mediated immunity in STAD, we also use bioinformatics analysis. In our study, the ASCL2 reveals a positive correlation with the levels of M2 macrophages and T follicular helper cells and shows a negative correlation with the levels of neutrophils, dendritic cells, monocytes, CD8 T cells, and M1 macrophages. Besides, the ASCL2 is revealed as a positive correlation for the levels of CD206 and CD109 and shows a negative correlation for the levels of MPO, CD1c, CD163, CD8a, and iNOS. All these data show that the ASCL2 may be involved in cell-mediated immunity in STAD.

Lymphocytes and macrophages produce ILs as pro-inflammatory cytokines to induce apoptosis of tumor cells and regulate the progression of tumors [21]. NF-κB modulates the expression and activity of DNA methyltransferases, histone modifiers, and miRNA; regulates inflammation; and activates pro-inflammatory cytokines, such as IL-1β and IL-18 [21]. TLR4 is the upstream regulator of the NF-κB signaling pathway [22]. TLR4 promotes the release of cytokines, chemokines, and costimulatory molecules which are proinflammatory transcription factors, playing a crucial role in inflammation [23–26]. Nevertheless, in STAD, the mechanism of ASCL2 in inflammation via TLR4 activation is not fully understood.

In our study, ASCL2 shows a negative correlation with *H. pylori* gastrointestinal inflammation by GSEA. The level of resistin, which is a regulator of inflammation, activates TLR signaling and toll-like receptor 4 cascade in STAD and is positively correlated with gastric cancer. In our study, sparstolonin B, as a TLR4 activator to stimulate TLR4 expression, is used to investigate the relationship between the ASCL2 and TLR4. Our results show that activation of TLR4 could regulate the level of downstream factors, after sh-ASCL2 administration. Furthermore, sparstolonin B seems to not be able to significantly alter the level of ASCL2, which may suggest that TLR4 may be the downstream factor of ASCL2. This part of our results demonstrates that the ASCL2 may be the upstream target factor of TLR4 and may possibly adjust inflammation through TLR4 activation in STAD.

Further bioinformatics analysis of the ASCL2 in STAD patients using the cBioPortal and GEPIA system shows that the ASCL2 may be related to *CNTNAP3*, *CLIP1*, *C9orf84*, *ARIH2*, and *IL1R2* mutations and involved in stomach carcinoma prognosis, which requires further verification in future studies.

## Conclusions

In this study, we indicate that the ASCL2 inhibition could significantly activate inflammation, weakening the development of tumor cells, and decreasing the level of biomarkers of gastric tumor through TLR4 activation. Taken together, these findings may offer new strategies for regulating inflammation in stomach carcinoma.

## Data Availability

The datasets used and/or analyzed during the current study are available from the corresponding author on reasonable request.

## Abbreviations

ASCL2: Achaete-scute complex-like 2
STAD: Stomach adenocarcinoma
TLR4: Toll-like receptor 4
